# Synthesis of silver nanoparticles using living electroactive biofilm protected by polydopamine

**DOI:** 10.1016/j.isci.2021.102933

**Published:** 2021-07-31

**Authors:** Yarui Liu, Xuemei Zhu, Qian Zhao, Xuejun Yan, Qing Du, Nan Li, Chengmei Liao, Xin Wang

**Affiliations:** 1 MOE Key Laboratory of Pollution Processes and Environmental Criteria, Tianjin Key Laboratory of Environmental Remediation and Pollution Control, College of Environmental Science and Engineering, Nankai University, No. 38 Tongyan Road, Jinnan District, Tianjin 300350, China; 2 School of Environmental Science and Engineering, Tianjin University, No. 92 Weijin Road, Nankai District, Tianjin 300072, China

**Keywords:** Electrochemistry, Microbiofilms, nanoparticles

## Abstract

The biosynthesis of metal nanoparticles from precious metals has been of wide concern. Their antibacterial activity is a main bottleneck restricting the bacterial activity and reduction performance. Here, bio-electrochemical systems were used to harvest electroactive biofilms (EABs), where bacteria were naturally protected by extracellular polymeric substances to keep activity. The biofilm was further encapsulated with polydopamine (PDA) as additional shield. Silver nanoparticles (AgNPs) were biosynthesized on EABs, whose electroactivity could be fully recovered after Ag^+^ reduction. The PDA increased bacterial viability by 90%–105%, confirmed as an effective protection against antibacterial activity of Ag^+^/AgNPs. The biosynthetic process changed the component and function of the microbial community, shifting from bacterial Fe reduction to archaeal methanogenesis. These results demonstrated that the electrochemical acclimation of EABs and encapsulation with PDA were effective protective measures during the biosynthesis of AgNPs. These approaches have a bright future in the green synthesis of nanomaterials, biotoxic wastewater treatment, and sustainable bio-catalysis.

## Highlights

• The EABs formed using BESs had an efficient ability to recover Ag^+^ to AgNPs
• The bio-reduction efficiencies of AgNPs reached more than 94%
• The PDA increased by 90%–105% of the bacterial viability
• The biosynthesis process changed the microbial community

## Graphical abstract

**Figure:**
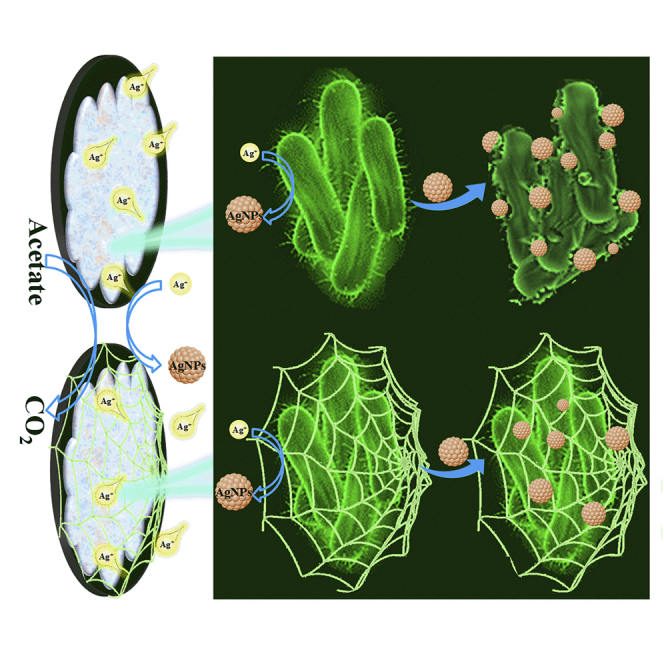

## Introduction

The use of Ag and Ag salts is as old as human civilization; they have been used as an effective antibacterial material for centuries. With the rapid expansion of modern industry, the Ag^+^ from electroplating wastewater could incur an ecological risk through enrichment along food chains if not well treated before discharging, because it could not be thoroughly degraded like organic pollutants (Eckelman and Graedel 2007). As a precious metal, the recovery of Ag has more benefit ecologically and economically than its removal. Reducing Ag ion to Ag nanoparticles (AgNPs) is one of the most effective and value-added methods for recovery (Vance et al., 2015), and the first step of recovery is the synthesis of AgNPs.

As an environmentally green process, the biosynthesis of metal nanoparticles (NPs) has been explored in recent years (Kong and Liu 2020; Shivaji et al., 2011), and it relies on the respiration of living microorganisms (Byrne et al., 2011). Conventional physical and chemical methods for the synthesis of NPs often require harsh conditions, which are usually costly and environmentally hazardous. The biosynthetic processes for the synthesis of NPs are more economical and environment-friendly protocols at ambient temperature and pressure without the addition of additional reagents. Many bacteria have been isolated and demonstrated to synthesize NPs, either extracellularly by the reduction of metal ions on the cell surface (Law et al., 2008) or intracellularly through the encapsulation of metal ions inside cells (Das et al., 2014). Extracellular synthesis favors large-scale production and requires simpler downstream processing than intracellular method, indicating a promising perspective on real applications. For example, the Fe(III)-reducing bacteria of *Geobacter* sp. possessed a surprisingly significant potential for extracellular reduction of Ag^+^ to AgNPs (Law et al., 2008). In this process, the electrons required for metal reduction are from substrate (such as organic wastes) oxidation. Then, they are transferred from the microbial endomembrane through the mutual transformation of Fe^2+^/hemes and Fe^3+^/hemes in cytochromes to the outer membrane. Finally, the Ag^+^ is reduced to AgNPs. These functional bacteria were also reported to transfer electrons to a solid electrode, namely, exoelectrogens (Esteve-Nuiiez et al., 2011).

For the extracellular synthesis of AgNPs, obtaining sufficient metal-reducing bacteria, such as exoelectrogens, is the first challenge owing to their strict growth condition, and keeping them dominant in the microbial community after long-term succession is difficult (Niu et al., 2021). Bio-electrochemical systems (BESs) were reported to select exoelectrogens from various environments (sediment, soil, or wastewater) in a couple of days and be stabilized for years, thus providing an applicable and simple method to collect and stabilize exoelectrogens (Li et al., 2020a). The second challenge is the biotoxicity of AgNPs. Different from other precious metal NPs, such as Pt or Au, AgNPs were found to be an excellent antimicrobial agent even in solid state (Siddiqi and Husen 2016). Thus, the newly formed AgNPs and the Ag^+^ in the solution could stop the microbial metabolism and inhibit the synthesis. Previous studies have focused on suspended exoelectrogens for the reduction of Ag^+^ to AgNPs (Law et al., 2008; Vasylevskyi et al., 2017). However, microorganisms have higher tolerance toward AgNPs in the form of biofilm than planktonic cells (Sheng and Liu 2017) owing to the protective effect of the accumulation of extracellular polymeric substances (EPSs) on the biofilm (Chen et al., 2022; Sheng et al., 2015). Coincidentally, BESs could form dense electroactive biofilms (EABs) on the electrodes during the process of enrichment of exoelectrogens, which acted as a natural protection. In 2017, a biocompatible natural material polydopamine (PDA) with comparable function as EPSs that could provide an additional protective shield on the basis of EPSs to help exoelectrogens survive in harsh environment was reported (Du et al., 2017). Therefore, the present study aimed to screen metal-reducing bacteria by BESs to form EABs and wrap them with a PDA protective shield to possibly solve both problems at the same time. The AgNPs attached to the cell surface or spatially embedded in EPSs could be interconnected (Zakaria et al., 2018). Thus, they may further act together with exoelectrogens as a living electrocatalyst for bioenergy production or the removal of refractory contaminants.

As a proof of concept, single-chambered BESs were used in this study to enrich metal-reducing bacteria to reduce Ag^+^ into AgNPs. PDA was tested as a protective shell to see whether the exoelectrogens were able to survive after AgNPs formation, and the mechanisms were discussed in accordance with the electrochemical performance, AgNPs morphology, spatial distribution of microbial activity, and succession of microbial communities.

## Results

### Electrochemical performance of BESs

**Figure 1:**
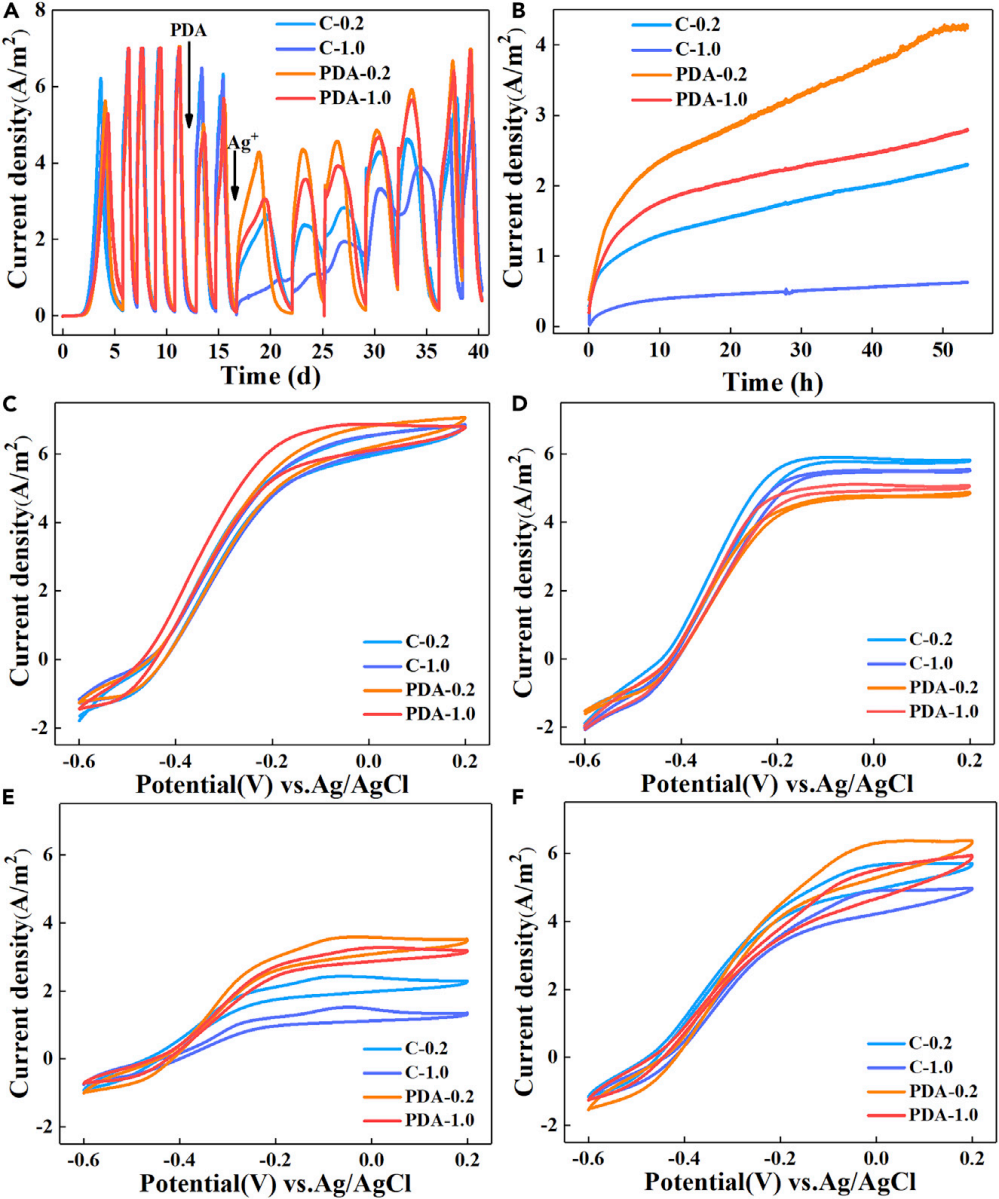
The current density and Cyclic voltammetrys (CVs) of BESs in different treatment processes The reference electrode was Ag/AgCl (4.0 M KCl, 0.201 V versus SHE). (A) The current density of BESs during all processes including domestication of mature electroactive biofilm, polydopamine encapsulation, and Ag^+^ bioreduction. (B) The current recovery at the first cycle of Ag^+^ reduction. (C) CVs of mature biofilms. (D) CVs of PDA-encapsulated biofilms. (E) CVs of biofilms in the prophase after Ag^+^ reduction. (F) CVs of biofilms in the recovery anaphase after Ag^+^ reduction.

A similar current density of 7.0 ± 0.03 A/m^2^ was observed in all BESs after five cycles of domestication (Figures 1A and S1A), indicating that the electroactivity of EABs in different reactors was highly repeatable. The encapsulation of PDA decreased the current density in all BESs, probably owing to the short-term introduction of oxygen during dopamine polymerization. In particular, the maximum current density of PDA-encapsulated anodes (PDA-) was reduced by 30% to 4.91 ± 0.11 A/m^2^, whereas that of the control without PDA (C-) marginally decreased by 8% (6.41 ± 0.08 A/m^2^). After two cycles of biofilm restoration, they were separately inserted into solutions containing 0.2 or 1.0 g/L Ag^+^ solution for AgNPs synthesis. As predicted, the bactericidal efficacy of Ag^+^ sharply decreased the current densities in all BESs, but that of Ag^+^ with PDA encapsulation started to recover after 0.5 h (Figure 1B). The current densities of PDA-0.2 were restored to 4.29 ± 0.08 A/m^2^, 1.6 and 4.4 times higher than those of C-0.2 (2.65 ± 0.07 A/m^2^) and C-1.0 (0.98 ± 0.1 A/m^2^) in 50 h. The feeding cycle of 1 g/L acetate was extended from 2 to 5 days, partly supporting that the biotoxic Ag^+^ inhibited the metabolic activity of exoelectrogens. At the end of the seventh cycle after Ag^+^ reduction, the current densities of the PDA-encapsulated biofilms returned to 6.96 ± 0.02 A/m^2^, almost fully recovered, whereas the no PDA controls still had 13% (C-0.2, 6.11 ± 0.06 A/m^2^) and 26% (C-1.0, 5.18 ± 0.03 A/m^2^) loss in current densities (Figure 1A).

The sigmoidal catalytic waves in CVs showed that the limiting current changed after each treatment but the curve shape did not (Figures 1C–1F). The main pair of symmetrical peaks in DCVs stabilized at −0.39 ± 0.01 V (Figure S2), suggesting that the dominant redox pair was not altered during the process of PDA encapsulation or Ag^+^ bio-reduction. The limiting current densities decreased, which was consistent with the results of chronoamperometry. Of interest, an irreversible oxidation peak at −0.11 ± 0.03 V was found in DCVs after Ag^+^ exposure, and it could be a result of the interaction between Ag^+^ and microorganisms.

### Bio-reduction of Ag^+^ to AgNPs

**Figure 2:**
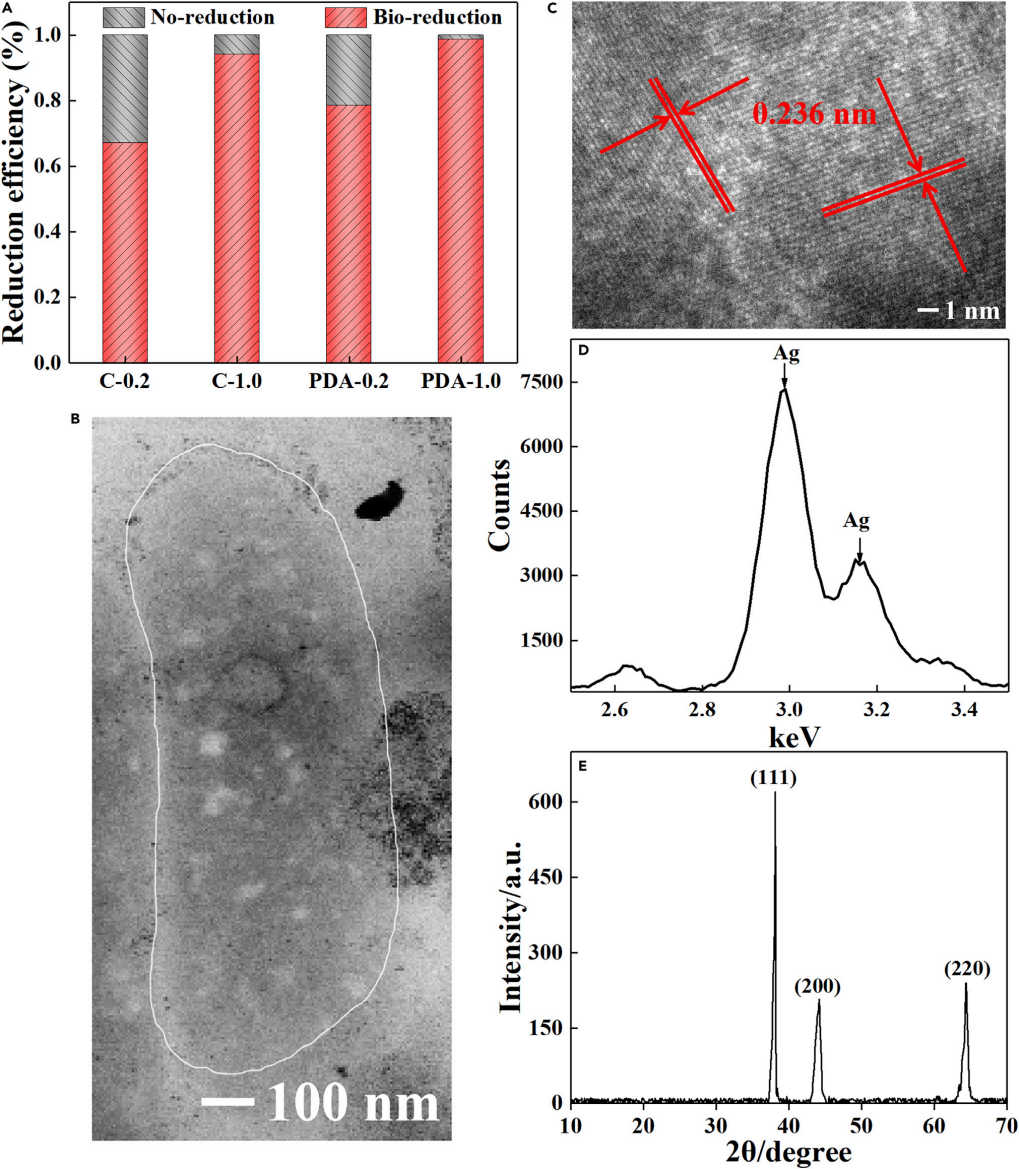
Structure characterization and spectral identification of AgNPs (A) The Ag^+^ removal efficiencies in solution. (B) Transmission electron microscopy (TEM) images of rod-like bacteria with AgNPs deposited on the surface. (C) The high-resolution TEM images of AgNPs. (D) The high-resolution TEM images, EDS of AgNPs. (E) The spectra, and typical XRD pattern of AgNPs.

The color of the solution containing Ag^+^ changed from yellow to brown after EAB reduction, whereas that without EABs still remained yellow after 12 h (Figure S3). The removal efficiencies of Ag^+^ reached 94% (C-1.0) and 99% (PDA-1.0) in the solution containing 1.0 g/L Ag^+^ compared with the 67% and 79% removal efficiencies of C-0.2 and PDA-0.2 (Figure 2A). PDA encapsulation enhanced the bio-reduction efficiency by 18% (0.2 g/L) and 5% (1.0 g/L).

Spherical or polygonal NPs with a diameter of ~30 nm covered the bacterial cell after Ag^+^ reduction (Figure 2B). The lattice fringes with a spacing of 0.236 nm observed via high-resolution transmission electron microscopy (TEM) showed a face-centered cubic metal, and no particle had grain boundaries inside it (Figure 2C). Elemental composition analysis showed a strong Ag^0^ signal in the spectrum of the reduction products (Figure 2D). Furthermore, the X-ray diffraction (XRD) pattern displayed typical Ag^+^ peaks at 2θ values of 38.2°, 44.3°, and 64.5°, which corresponded to (111), (200), and (220) crystal planes with a calculated lattice constant of 4.086 Å, respectively, thus confirming that these NPs were Ag (Figure 2E) (Zhang et al., 2019).

**Figure 3:**
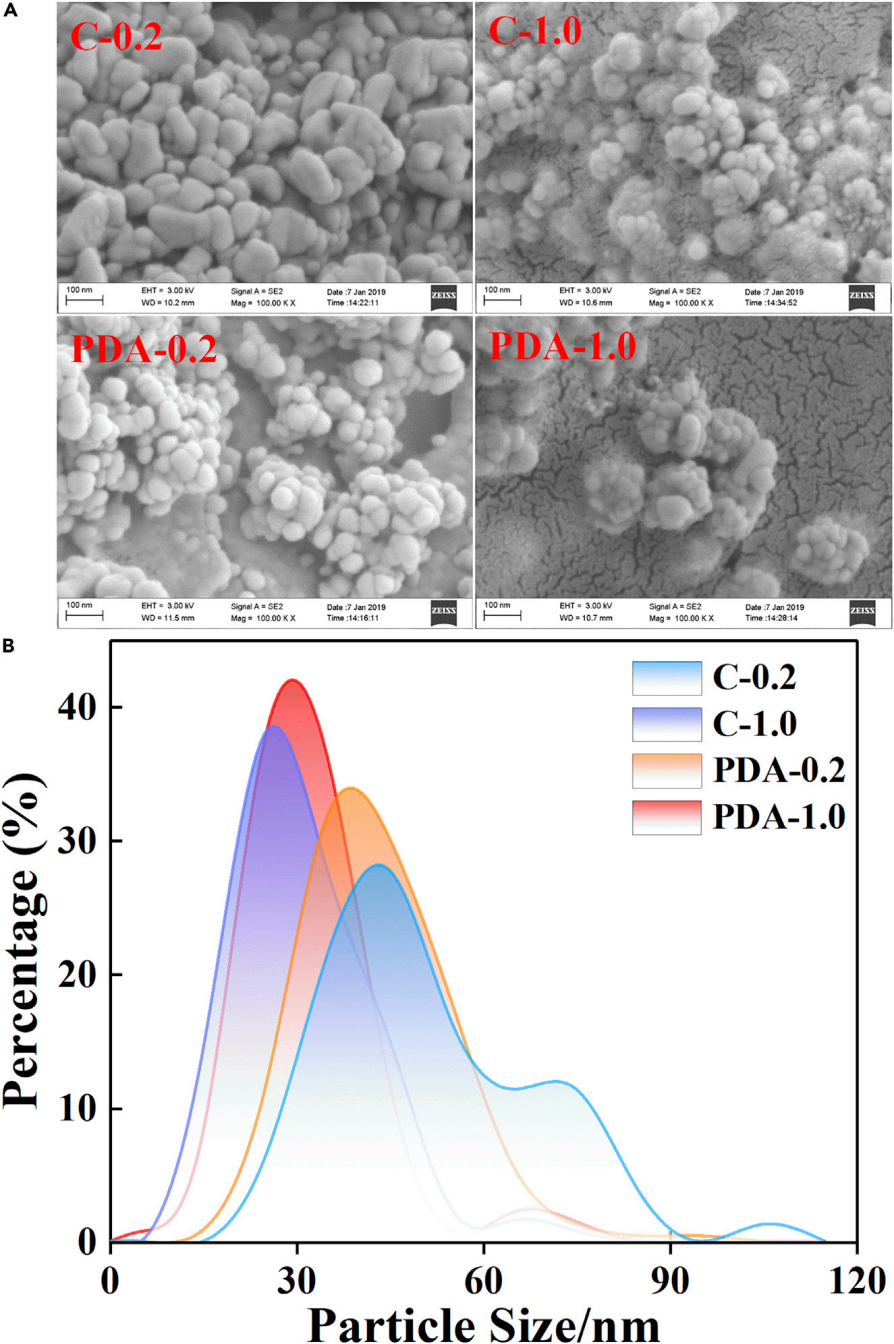
Scanning electron microscope (SEM) images and size distribution of AgNPs formed by the bioreduction of Ag^+^ (A) SEM images of AgNPs formed by the bioreduction of Ag^+^. (B) The size distribution of AgNPs formed by the bioreduction of Ag^+^.

Further research showed that most AgNPs were attached to the biofilm surface almost throughout the area investigated, where the cross-linking network composed of intensive rod-shaped bacterial cells and EPSs, giving rise to a unique interconnected rough and porous structure by combining with spherical AgNPs (Figure S4). More interestingly, the particle size and uniformity of the spherical AgNPs biosynthesized on the surface of EABs varied with the concentration of Ag^+^ (Figure 3). The NPs reduced in 1.0 g/L of Ag^+^ (mean ≈30 nm, 95% concentrated at 10–50 nm) were smaller and more uniform than those in 0.2 g/L of Ag^+^ (mean ≈40 nm, 95% concentrated at 20–80 nm). No effect was observed on the particle size distribution of AgNPs, whether encapsulated with PDA or not (Figure S5).

### Viability and microbial community of EAB after Ag^+^ reduction

**Figure 4:**
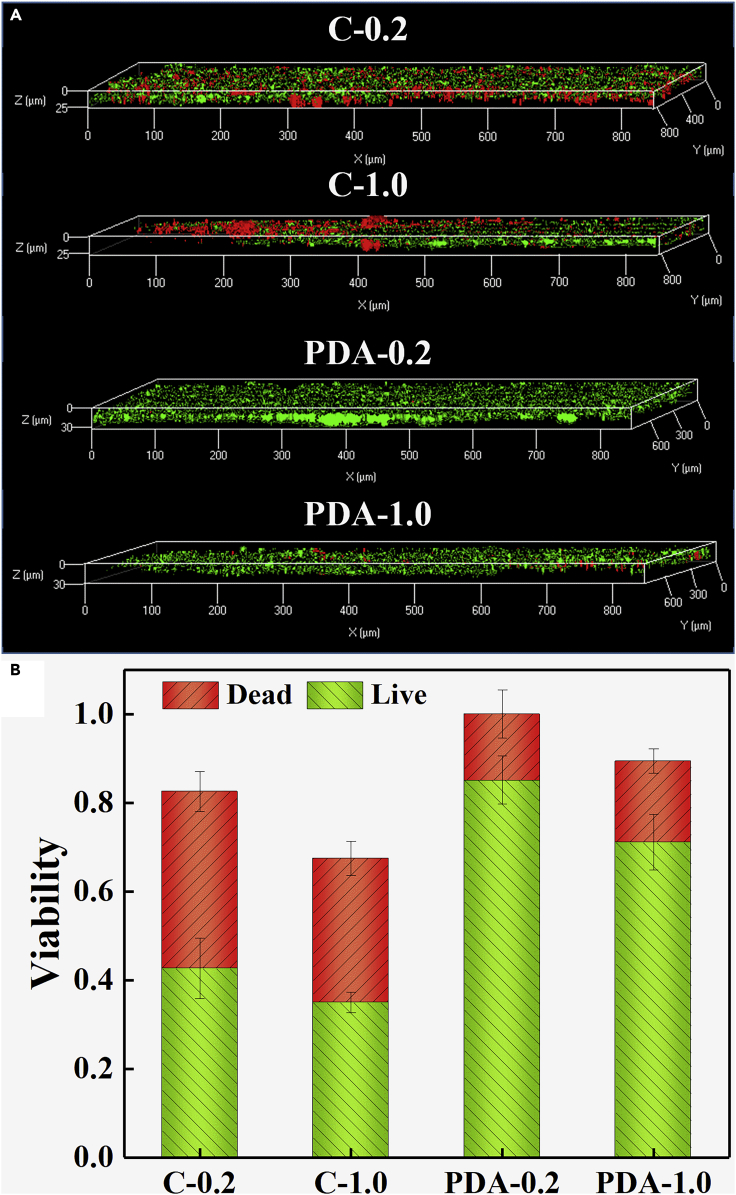
Viability of EAB after Ag^+^ reduction (A) Stacked CLSM images of biofilms after Ag^+^ shocks. The biofilm at 0 μm means the biofilm−electrode interface (bottom). (B) Viability is calculated by counting pixels. Error bars are derived from the calculation of two parallel samples.

The EABs changed from sticky pink to grainy black brown after Ag^+^ reduction (Figure S6), implying the adhesion of AgNPs and destruction of EABs activity. Those coated with PDA still had strong growth and reproduction activity after Ag^+^ reduction (Figure 4A), with biofilms 1.3 (PDA-0.2, 36 μm) and 1.1 (PDA-1.0, 32 μm) times thicker than those of the control (C-0.2, 28 μm; C-1.0, 30 μm) and more viable cells (Figure S7). Further calculation and normalization (Du et al., 2017) on the dead/alive ratio of EABs showed that the viabilities in PDA-encapsulated biofilms were up to 0.88 (PDA-0.2) and 0.74 (PDA-1.0), which were 90% and 105% higher than those in the corresponding control (C-0.2, 0.46; C-1.0, 0.36). According to the statistical analysis (Table S1), the viabilities in PDA-encapsulated EABs was significantly different from the control, with greater resistance (Figure 4B). These findings demonstrated the protection of PDA on cell activity against toxic Ag^+^ and AgNPs.

The composition, function, and symbiotic interrelation of the microbial community could directly reflect the interaction between microorganisms in the biosynthesis of AgNPs. According to the species annotations, Proteobacteria (83%–93%) predominated in the mature EABs as previously described (Li et al., 2020b). However, the percentages of this phylum substantially decreased to 26%–39% after exposure to Ag^+^, especially under high Ag^+^ concentration, whereas Euryarchaeota and Bacteroidetes correspondingly increased from 2.5% to 23% and 7.6%–26%, respectively (Figure S8).

**Figure 5:**
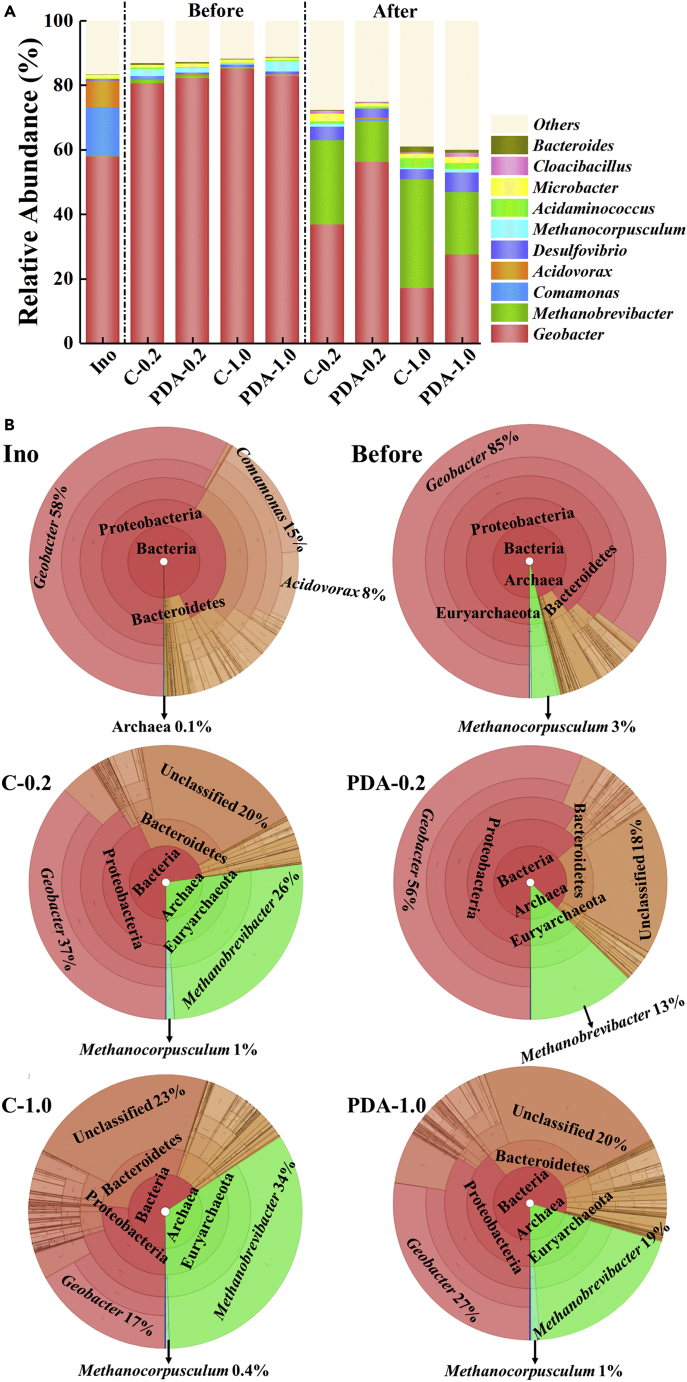
The microbial community of EAB at genus level. “Before” and “after” denote the biofilm samples before and after Ag^+^ reduction, respectively (A) Relative abundance of bacterial communities at genus level. (B) Krona analysis of bacterial community in the process of Ag^+^ reduction.

The detailed compositions at genus levels revealed that *Geobacter* dominated in the mature EABs, demonstrating its main force status of reducing Ag^+^ to AgNPs. However, its abundance lessened evidently along with the increase in *Methanobrevibacter* after Ag^+^ bio-reduction. With C-1.0 as an example, the percentage of typical exoelectrogens, *Geobacter*, belonging to bacteria decreased from 85% to 17%, whereas the *Methanobrevibacter* belonging to archaea increased from 0.43% to 34%. Selection of two dominant domains of bacteria and archaea for Krona analysis showed that the proportion between archaea (mainly *Methanobrevibacter*) and bacteria (mainly *Geobacter*) increased significantly with the toxic stress from Ag^+^ or AgNPs. The EABs with PDA protection clearly reduced this toxic stress, further demonstrating the protective function of PDA (Figure 5B). The proportion of *Geobacter* in EABs with PDA protection was apparently higher than that without PDA shield. The phylogenetic relationship of species at the genus level also showed that *Methanobrevibacter* was not closely related to *Geobacter*, thus suggesting different tolerance to toxicity (Figure S9). The unique role of other genera in Ag^+^ reduction has not yet been specifically studied.

**Figure 6:**
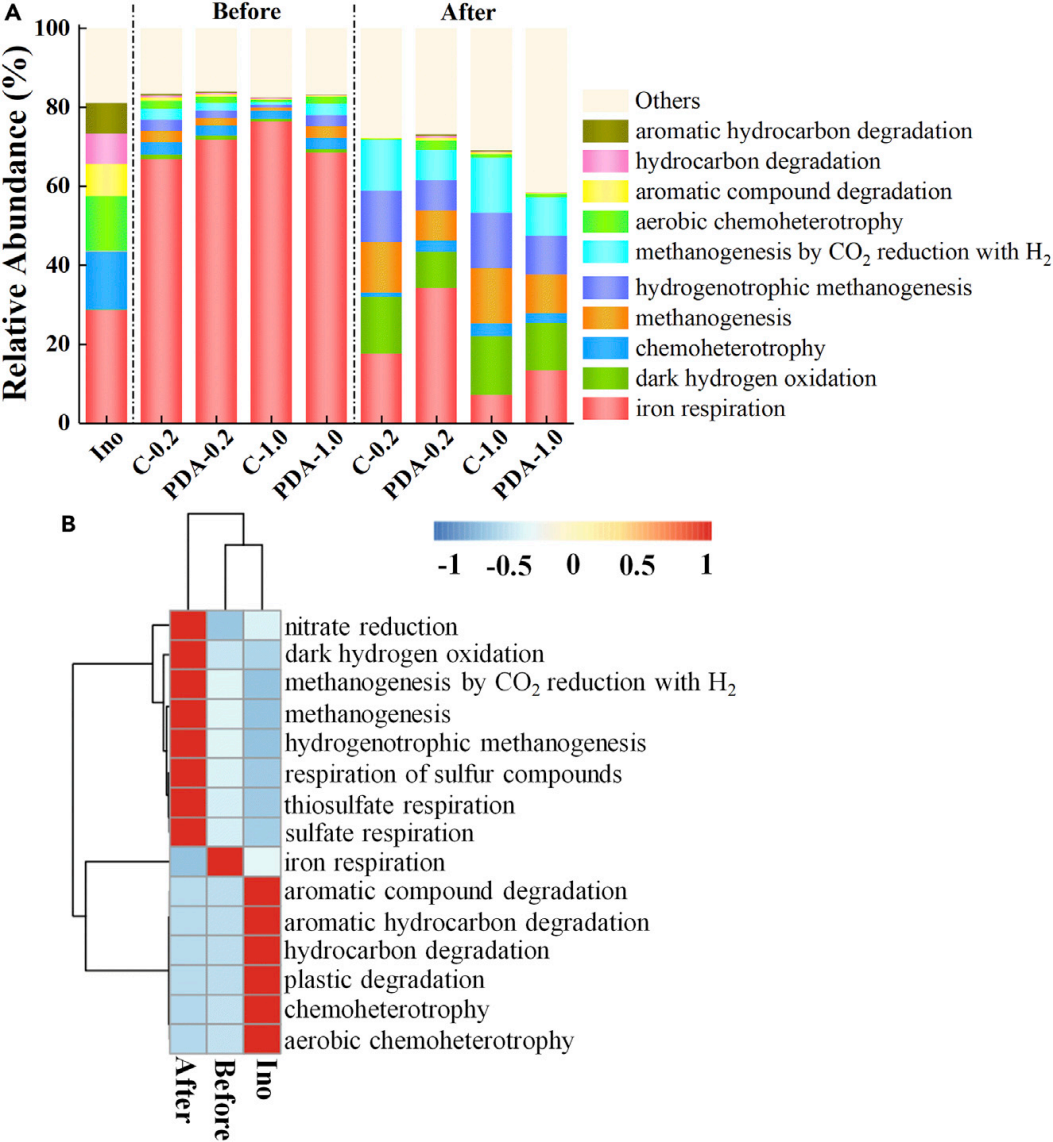
The relative abundance of functional information of bacterial community. “Before” and “after” denote the biofilm samples before and after Ag^+^ reduction, respectively. (A) The column plot of relative abundance of functional information of bacterial community. (B) The thermogram of relative abundance of functional information of bacterial community.

The function of the community derived from the microbial component showed that Fe respiration was the absolute leading function in mature EABs before Ag^+^ reduction (Figure 6). However, the main function sharply changed after Ag^+^ treatment. For instance, the functional abundance of Fe respiration in C-1.0 decreased from 76% to only 6.9%, whereas the proportion of functions related to hydrogenotrophic methanogenesis increased by 19 times (Figure 6A). As illustrated by the functional thermogram in Figure 6B, the acclimation of microbes in BESs remarkably changed their function from organic degradation to iron respiration, while the reduction in Ag^+^ further switched the function to methanogenesis.

The symbiotic network of bacteria and archaea characterized the adaptability of microorganisms and the interaction of dominant species. The degree of association between species in biofilm extensively became concise and explicit after Ag^+^ reduction. In particular, the interaction among bacteria (such as *Comamonas*, *Acidovorax*, *Bacteroides*, *Pseudomonas*, and *Rhodocyclaceae*) seemed to be broken. In addition, the negative correlation focusing on electroactive *Geobacter* and hydrogenotrophic methanogenesis was not altered with the upheaval of Ag^+^/AgNPs, which also corresponded to the change in microbial community compositions and function.

## Discussion

Stable currents were obtained in BESs after five cycles of domestication (Figure S1), indicating the successful enrichment of exoelectrogens (Law et al., 2008; Vasylevskyi et al., 2017). The color of Ag^+^ solution varied from yellow to brown after EABs addition (Ahmad et al., 2003), preliminarily indicating Ag^+^ bio-reduction (Figure S3). Material analysis confirmed the spherical or polygonal AgNPs closely bonded to bacterial cells, ranging from 10 to 110 nm, which was from the reduction of EABs. The reduction of Ag^+^ to Ag^0^ was a highly endergonic reaction step; only the rapid, exergonic aggregation to Ag_n_ clusters led to the successful synthesis of AgNPs. Thus, a steady electron flux was a critical requirement favoring AgNP formation using EABs. The high current of BESs in the closed-circuit state expounded that the anodic exoelectrogens maintained the extracellular electron transport flux with high efficiency. When the circuit was switched to open state, Ag^+^ acted as an electron acceptor instead of cathode forming AgNPs. In this process, the microbes belonging to *Geobacter* were more than 85% in abundance, providing reducing force through microbial metabolism. This finding was in accordance with the community function shown in Figure 6, where Fe respiration dominated the microbial community.

Unfortunately, Ag^+^ and AgNPs are highly biotoxic for bacterial strains (Siddiqi et al., 2018), which played a persecuting role at cellular, subcellular, and biomolecular levels (Jayasree et al., 2010). In particular, given that Ag^+^ ions are positively charged and much smaller than neutral AgNPs in size, they could easily penetrate into the microbial cell by interacting with electron-rich biomolecules in the cytoderm containing S or P and N via diffusion, leading to the denaturation of protein and cell death (Hamouda et al., 2001). Although the EABs quickly reduced Ag^+^ to AgNPs, which abated the cytotoxicity, the long-term existence of AgNPs with antibacterial activity remained a hidden trouble to cellular activity. The NPs synthesized at a high concentration of Ag^+^ were smaller than those synthesized at a low concentration (Figure 3). In addition, smaller AgNPs induced greater cytotoxicity because the increased specific surface area provided more interactions with the microbial cells (Chwalibog et al., 2010). Thus, the EABs reducing 1.0 g/L Ag^+^ suffered from stresses from small AgNP size and large Ag^+^ concentration, thereby reducing the current density and bacterial viability and changing the microbial community.

**Figure 7:**
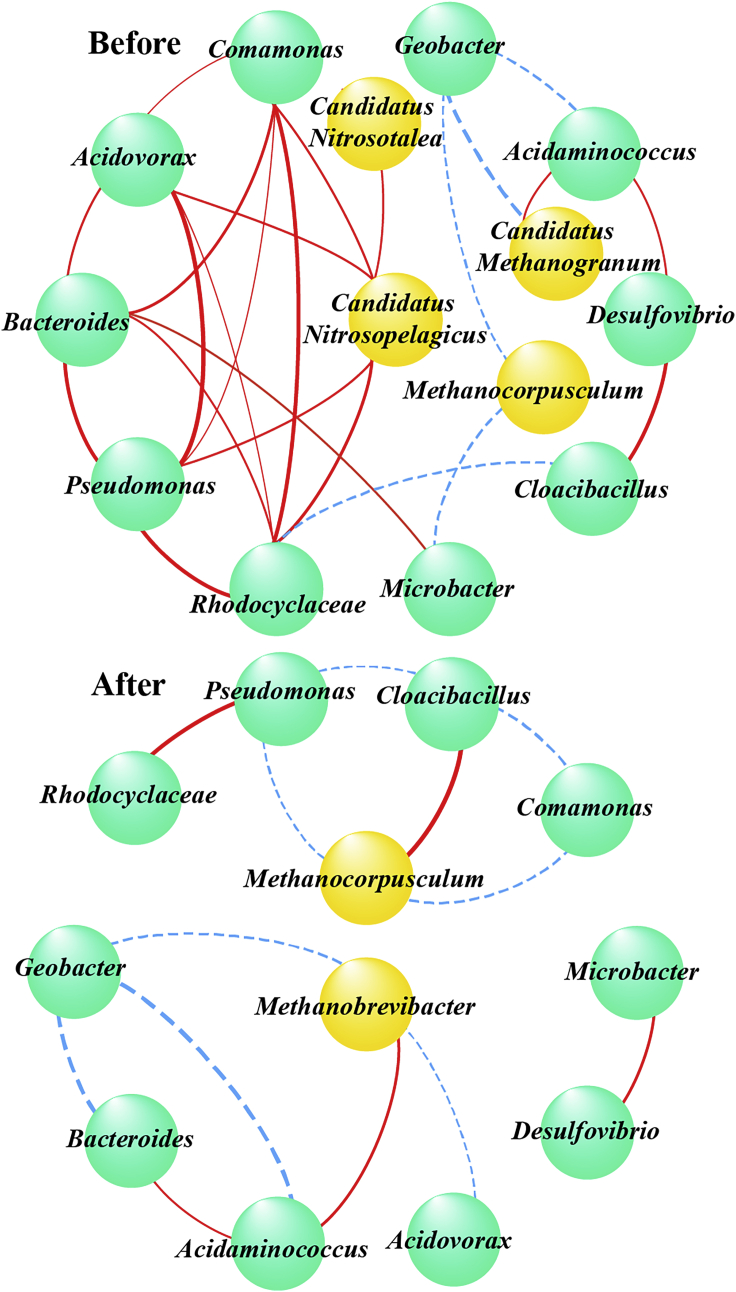
The symbiotic network of correlation coefficient at genus level The correlation coefficient of effective connection was greater than 0.6 or less than - 0.6 and significant (p value less than 0.05). Green is bacteria, yellow is archaea, red represents positive correlation, blue dotted line represents negative correlation, and the thickness of the line represents the size of the correlation. “Before” and “after” denote the biofilm samples before and after Ag^+^ reduction, respectively.

The component, function, and symbiotic interrelation of microbial community remarkably changed after Ag^+^ bio-reduction (Figures 5, 6, and 7). At the stage of EABs acclimation, the *Geobacter* belonging to electroactive bacteria with very suitable growing environment was far more competitive than the *Methanobrevibacter* belonging to hydrogenotrophic archaea, leading to its absolute dominance in EABs. However, short-term Ag^+^ exposure and long-term AgNPs stimulation annihilated a part of *Geobacter* but hardly persecuted *Methanobrevibacter*, resulting in the gradual succession from bacteria to archaea. This phenomenon might be attributed to the relatively thin cell membrane of Gram-negative *Geobacter* with poor resistance to AgNPs toxicity (Sondi and Salopek-Sondi 2004). The outer layer of Gram-negative bacteria was made up of a lipopolysaccharide layer, and the inner layer was composed of a linear polysaccharide chain forming a three-dimensional network with peptides. The NPs synthesized on the surface of EABs formed pits in the cell wall of microorganisms accumulated and permeated into the bacterial cell, whose adsorption and penetration hindered cell respiration, prevented cell replication, and eventually led to bacterial death (Tho et al., 2013). However, methanogenic archaea were demonstrated to be tolerant to AgNPs (Yang et al., 2012; Yu et al., 2012). This succession of microbial community switched the main function from Ag^+^ bio-reduction to the archaeal resistance to AgNPs (Figures 6 and 7).

The accumulation of abundant EPSs throughout EABs (Figure S4) could protect cells from germicides (Ag^+^ and AgNPs) and whose various ligands could also reduce Ag^+^ and immobilize AgNPs (Kang et al., 2014). Therefore, harvesting EABs with BESs could resist Ag^+^ even at concentrations up to 1.0 g/L, which was 46 times higher than the 0.2 mM (0.022 g/L) Ag^+^ reduced by suspension bacteria (Law et al., 2008). In addition to the natural protective effect of EPSs, an important measure was to wrap the PDA outside the EABs to weaken the invasion of germicide. The high-stability PDA with good biocompatibility and abundant functional groups could closely combine with bacterial cells and form a lasting solid barrier wrapping cells, resisting the deposition and penetration of AgNPs. The extracellular electron transfer ability of the exoelectrogens was well protected by PDA, as evidenced by their much higher current density than the control, whether the concentration of Ag^+^ was 0.2 or 1.0 g/L (Figure 1). In particular, during the first recovery cycle after Ag^+^ reduction, the EABs packed in PDA shield were able to rapidly resume their metabolic activity (current density) as soon as the extreme conditions were eliminated and fully recover activity after seven cycles of convalescence, thus exhibiting stronger reusability rather than disposable products. A more direct evidence was the livability of microorganisms in EABs, where the protection by PDA was enhanced by 2.1-fold over that of pure EPSs (Figure 4). These data were from a single Ag^+^ reduction. After multiple high toxic exposures, EABs without the PDA protective shield may suffer from an irrecoverable collapse. The succession between low-tolerance *Geobacter* and high-tolerance *Methanobrevibacter*, including abundance and function, became another indicator of microbial community invasion by germicide in EABs, showing a trend of C-1.0 > C-0.2 > PDA-1.0 > PDA-0.2. This finding was in full agreement with the electroactivity and survival of EABs, thus fully confirming that PDA is an indispensable measure in the technology of AgPs biosynthesis recovery by EABs.

In this study, EABs were formed by the aggregation of microorganisms using BESs, and Ag^+^ was recovered in the form of AgNPs by the unique extracellular electron transfer ability of exoelectrogens. PDA was used to wrap EABs, forming a protective shield to better resist the extreme persecution of Ag^+^ and AgNPs. The results showed that EABs had an efficient ability to recover Ag^+^ and PDA possessed a strong protective effect. After a recovery period, the activity of PDA-encapsulated EABs could be fully restored, showing that the technology was not a disposable product. After one stimulation, the microorganisms did not develop resistance to Ag^+^. However, as they increased their resistance feedback to unfavorable environmental factors, the rehabilitation cycle after subsequent Ag^+^ reduction could be considerably shortened, which is an incomparable advantage of other technologies. This study offered a method for synthesis Ag^+^ as potentially useful AgNPs and using effective PDA protection measures against the biotoxicity in the Ag^+^ reduction process. This technology is simple, cost-effective, and eco-friendly, and it could be easily scaled up for high yields and/or production. It also has promising applications in the green synthesis of nanomaterials, biotoxic wastewater treatment, and sustainable bio-catalysis.

### Limitations of the study

A notable detail is that the electroactive biofilm community in this study was primarily composed of various exoelectrogens species because BESs were inoculated with high abundance of electroactive bacteria and operated in acetate medium. The biofilm community of BESs was expected to be highly diverse owing to a huge variety of organic substances during treatment of real wastewater. Hydrolysis and subsequent fermentation of complex organics were the rate-limiting steps for the current generation from BESs fed with real wastewater having particulate organics and various fermentable substrates. The syntrophic interactions of exoelectrogens with hydrolytic and/or fermentative bacteria could be crucial. Thus, further research is needed to examine the toxicity of AgNPs on the potential syntrophic of electroactive bacteria if this technology was applied to actual water. Furthermore, the biosynthesis rate and stability of AgNPs were an important part of industrial production. Therefore, monitoring the reaction conditions properly is also important. Biosynthesis of nanoparticles is only the first step to realize the recovery of precious metals, and the subsequent purification and characterization are also the key steps, so a lot of research is needed to explore in order to achieve practical application.

## STAR★Methods

### Key resources table

**Table:** 

REAGENT or RESOURCE	SOURCE	IDENTIFIER
**Biological samples**		

Electroactive microorganism	Our laboratory	This study

**Chemicals, peptides, and recombinant proteins**		

LIVE/DEAD BacLight bacterial viability kit	Thermo Fisher Scientific Inc.	L13152
Soil genomic DNA kit	Com Win Biotech Co. Ltd., Beijing, China	CW2091S

**Critical commercial assays**		

Illumina MiSeq	Novogene	V4 region of the 16S rRNA gene

**Deposited data**		

All sequence data	NCBI/Sequence Read Archive (SRA)	PRJNA721656

**Software and algorithms**		

ZEN black software	Airyscan, Zeiss, Germany	CLSM, LSM880
ZEN blue software	Airyscan, Zeiss, Germany	CLSM, LSM880

### Resource availability

#### Lead contact

Further information and requests for resources and reagents should be directed to and will be fulfilled by the lead contact, Dr. Chengmei Liao, lchmei@nankai.edu.cn.

#### Materials availability

This study did not generate nor use any new or unique reagents.

This study did not generate new unique reagents.

#### Data and code availability

Genomic DNA sequence data of biofilm have been deposited at NCBI/Sequence Read Archive (SRA) and are publicly available as of the date of publication. Accession numbers are listed in the key resources table. Microscopy data reported in this paper will be shared by the lead contact upon request. This paper does not report original code. Any additional information required to reanalyze the data reported in this paper is available from the lead contact upon request.

### Experimental model and subject details

A mixed culture of microorganisms obtained from microbial fuel cells fed with acetate (operated over 2 years) in the laboratory. The culture medium was 50 mM phosphate buffer solution (PBS, Na_2_HPO_4_, 4.576 g/L; NaH_2_PO_4_, 2.132 g/L; NH_4_Cl, 0.31 g/L; and KCl, 0.13 g/L), 12.5 mL/L trace mineral, 5 mL/L vitamin solution, and 1 g/L sodium acetate. The culture temperature was 25°C ± 1°C.

### Method details

#### Bioreactor configuration and operation

Three-electrode single-chamber BESs were assembled as previously described (Li et al., 2017) to provide a stable redox environment at the anodes. The reactor was framed by a cylindrical chamber (5 cm in length and 5 cm in diameter) with an effective volume of 100 mL, which was tightly sealed with a polytetrafluoroethylene cover to maintain the anaerobic environment. The working electrode (WE) was a graphite rod with an effective immersion area of 15 cm^2^. The counter electrode (CE) was a plain platinum plate (1 cm × 1 cm), and the reference electrode (RE) was Ag/AgCl (4.0 M KCl, 0.201 V versus standard hydrogen electrode); they were all purchased from Aida Hengsheng Technology Co., Ltd., Tianjin, China. As shown in Figure S10, three electrodes were connected to a multichannel potentiostat (CHI 1000C, Chenhua Instruments, Shanghai, China) poising the electrode potential at 0 V (vs Ag/AgCl). All potentials mentioned were referred to Ag/AgCl (4.0 M KCl) except as noted.

The reactors were inoculated with a mixed culture of microorganisms obtained from microbial fuel cells fed with acetate (operated over 2 years) in the laboratory. After the anodic biofilm was developed, the inoculum was switched to clean electrolyte containing 50 mM phosphate buffer solution (PBS, Na_2_HPO_4_, 4.576 g/L; NaH_2_PO_4_, 2.132 g/L; NH_4_Cl, 0.31 g/L; and KCl, 0.13 g/L), 12.5 mL/L trace mineral, 5 mL/L vitamin solution, and 1 g/L sodium acetate (Lovley and Phillips 1988). The electrolyte was refreshed and continuously bubbled with N_2_/CO_2_ (4:1) for 30 min to remove dissolved oxygen when the current was lower than 0.1 mA (approximately every 2 days), and this process was recorded as a cycle. All reactors were operated in batch mode at 25°C ± 1°C in a constant temperature incubator.

#### PDA encapsulation and bio-reduction of Ag^+^

The encapsulation of PDA referred to that in the previous study in the laboratory (Du et al., 2017). Anodes with mature electroactive biofilms (approximately five cycles) were immersed into 2 mg/mL dopamine solution prepared by dissolving dopamine hydrochloride in Tris-HCl buffer (10 mmol/L, pH 8.5) for 30 min to encapsulate the biofilm with PDA. Immediately after, the electrodes were thoroughly washed with PBS to remove residual lye. The anode without PDA encapsulation was prepared at the same time by immersing in Tris-HCl buffer for 30 min as control to compare the protective effect of PDA on anode microorganism.

After two cycles of biofilm restoration, the biofilm with/without PDA encapsulation were immersed into 0.2 g/L and 1.0 g/L Ag^+^ solutions, respectively. Each treatment was duplicated for 12 hr with the potentiostat disconnected. Then, the solution was replaced with fresh electrolyte, and the potentiostat was connected to record the current. Two parallel reactors were set up for different treatment groups.

#### Electrochemical Analyses

Current variations were recorded every 100 s by using chronoamperometry. The electrochemical activity of BESs were investigated using turnover cyclic voltammetry (CV) over a potential range of −0.6–0.2 V at a scan rate of 1 mV/s (stabilization period of 300 s. The first derivative CVs (DCVs) were calculated using the central difference quotient to further evaluate the changes in the peak height and midpoint potentials (Hong et al., 2011).

#### Characterization of AgNPs

An atomic absorption spectrometer (ContrAA 700, Analytik-JENA, Germany) was utilized to measure the residual Ag^+^ in the solution. The detailed structure and elemental composition of Ag^+^ reduction products were analyzed using transmission electron microscope coupled with energy dispersive spectroscopy (TEM-EDS, JEM-2800, JEOL, Japan). X-ray diffractometer (XRD, Ultima IV, Rigaku Corporation, Japan) was used to further determine the characteristic spectral peaks of the products. After the EABs were fixed overnight using 2.5% (wt/vol) glutaraldehyde and dehydrated with ethanol gradient (gradually increasing the concentration from 10% to 100%), scanning electron microscopy (SEM, MERLIN Compact, Zeiss, Germany) was employed to observe the surface morphologies on biofilms at an accelerating voltage of 10 kV. In brief, the morphology, detailed structure, and elemental composition of AgNPs were characterized by TEM-EDS, XRD, and SEM.

#### Spatial structure of EAB

Confocal laser scanning microscopy (CLSM, LSM880 with Airyscan, Zeiss, Germany) was used to image the activity space distribution of EAB. Biofilm-covered graphite sticks were cut with a sterilized blade and placed on an imaging dish filled with 50 mM PBS. The samples were dyed with the LIVE/DEAD BacLight Bacterial Viability Kit (L13152, Thermo Fisher Scientific Inc.) for 30 min, incubated in the dark, and then rinsed with 50 mM PBS to eliminate the excess dye before moving to CLSM. ZEN Black software was used to reconstruct 3D images. The percentages of viability and biofilm coverage were calculated using ZEN Blue software in accordance with counting pixels. Tamhane (T2) was used for statistical analysis by SPSS software. Tamhane T2 test is a non-parametric test for the analysis of sample variances that do not conform to the homogeneous distribution. Where the ∗ sign represents the presence of significant differences between the two groups of the test.

#### Biological analyses

The biofilm samples were collected by scraping graphite rods with the use of a sterile blade before and after reducing different concentrations of Ag^+^. Genomic DNA was extracted from the samples by using a Soil Genomic DNA Kit (CW2091S, Com Win Biotech Co. Ltd., Beijing, China) in accordance with the manufacturer's instructions. The microbial consortia were analyzed using Illumina MiSeq by Novogene (Beijing, China), with the universal primers including forward primer 515F (5′-GTGCCAGCMGCCGCGGTAA-3′) and reverse primer 806R (5′-GGACTACHVGGGTWTCTAAT-3′) to amplify the hypervariable V4 region of the 16S rRNA gene. The effective tags of all samples were clustered by operational taxonomic units with 97% identity to study the species composition of each sample, and then the OTU sequences were annotated.

The function prediction of tax4fun was realized by the nearest neighbor method based on the minimum 16S rRNA sequence similarity, whose specific method was to extract the whole-genome 16S rRNA gene sequence from KEGG database and use BLASTN algorithm to compare it to Silva SSU ref NR database (blast bitcore >1500) to establish the correlation matrix. The whole-genome function information of KEGG database annotated by uproc and pauda was mapped to Silva database to realize the function annotation of this database. OTUs were clustered on the basis of the sequence of Silva database to obtain functional annotation information.

The symbiotic network provided a new perspective for the study of community composition and the function of complex microbial environment. The symbiotic network could directly reflect the influence of different environmental factors on bacterial adaptability and the distribution of dominant species and closely interacting species in a certain environment, because the symbiotic relationship of microorganisms in different environments quite differ. These dominant species and species groups often play a unique and important role in maintaining the stability of microbial community composition and function in the environment. The symbiotic network of bacteria and archaea meeting specific parameters (correlation coefficient R > |0.6| and significant P < 0.05) was screened to characterize the adaptability of microorganisms and the interaction of dominant species to further explore the effect of AgNPs stimulation on the microbial community. R language was used for statistical tests and to visualize the results. All sequence datasets in this study were available at NCBI/Sequence Read Archive (SRA) under study with Bioproject database under the accession number PRJNA721656.

### Quantification and statistical analysis

ZEN Black software was used to reconstruct 3D images. The percentages of viability and biofilm coverage were calculated using ZEN Blue software in accordance with counting pixels. Tamhane (T2) was used for statistical analysis by SPSS software. Tamhane T2 test is a non-parametric test for the analysis of sample variances that do not conform to the homogeneous distribution. Where the ∗ sign represents the presence of significant differences between the two groups of the test. The analysis results are shown in Table S1.

### Additional resources

All sequence datasets in this study were available at NCBI/Sequence Read Archive (SRA) under study with Bioproject database under the accession number PRJNA721656, which can be accessed using the following link: https://dataview.ncbi.nlm.nih.gov/?search=SUB9469823&archive=bioproject.
