# Supplementary material for: Synthesis of silver nanoparticles using living electroactive biofilm protected by polydopamine
Source: iScience. 2021 Jul 31;24(8):102933. doi: 10.1016/j.isci.2021.102933 (PMC8361215; doi:10.1016/j.isci.2021.102933)
Supplement: Document S1. Figures S1–S10 and Table S1 [file mmc1.pdf]

iScience, Volume *24*

Supplemental information

Synthesis of silver nanoparticles using living electroactive biofilm protected by polydopamine

Yarui Liu, Xuemei Zhu, Qian Zhao, Xuejun Yan, Qing Du, Nan Li, Chengmei Liao, and Xin Wang

-Supporting Information-

Table S1 The significance testing of different EABs by T2. Related to Figure 4.

Group A	Group B	Mean difference	Significance testing
C-0.2	C-1.0	0.00837	1.000
	PDA-0.2	0.76109*	0.047
	PDA-1.0	0.67825*	0.029
C-1.0	C-0.2	−0.00837	1.000
	PDA-0.2	0.75272*	0.040
	PDA-1.0	0.66988*	0.026
PDA-0.2	C-0.2	−0.76109*	0.047
	C-1.0	−0.75272*	0.040
	PDA-1.0	−0.08283	0.799
PDA-1.0	C-0.2	−0.67825*	0.029
	C-1.0	−0.66988*	0.026
	PDA-0.2	0.08283	0.799

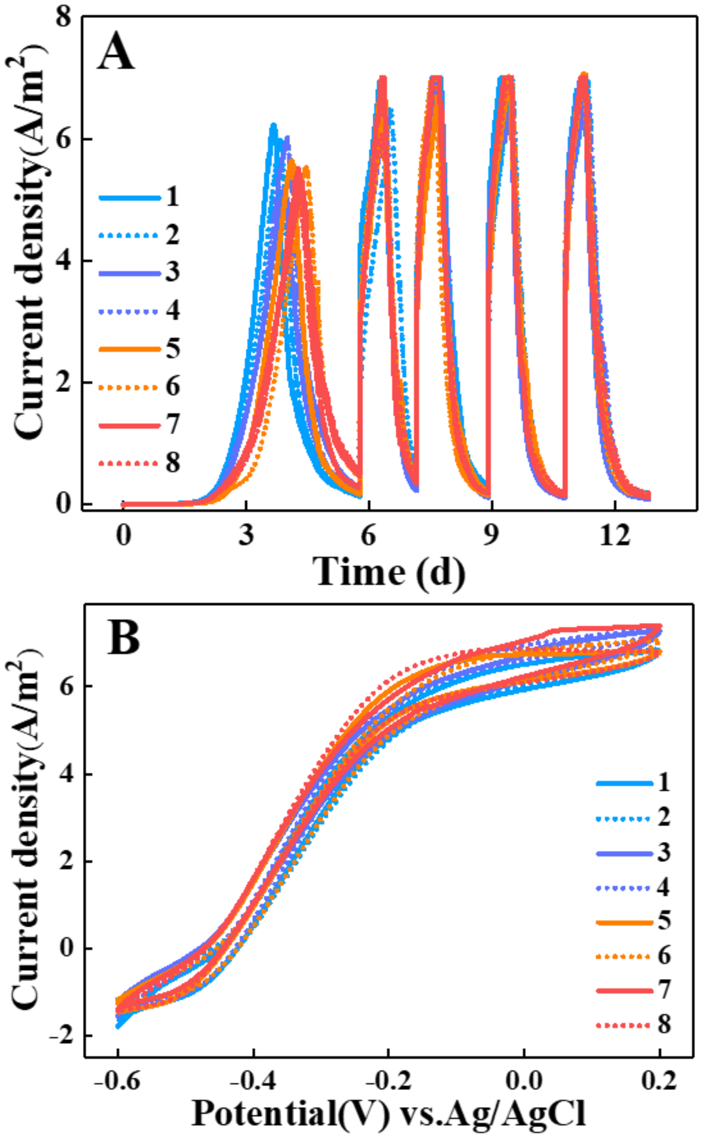

Figure S1. The current density (A) and CVs (B) of mature electroactive biofilm. The reference electrode was Ag/AgCl (4.0 M KCl, 0.201 V versus SHE). Solid and dashed lines represent two parallel reactors. Related to Figure 1.

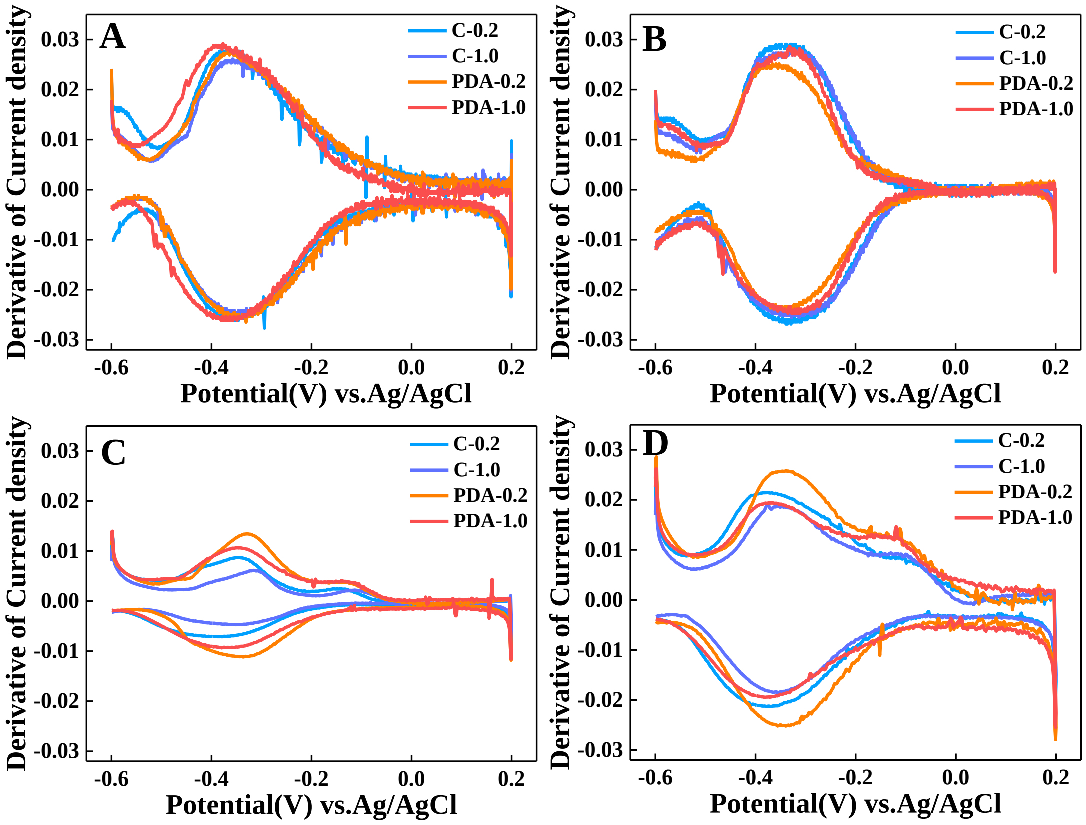

Figure S2. First derivative CVs (DCVs) of BESs in different treatment processes, including mature biofilms (A), PDA encapsulated biofilms (B), the prophase (C) and recovery anaphase (D) after Ag^+ reduction. The reference electrode was Ag/AgCl (4.0 M KCl, 0.201 V versus SHE). Related to Figure 1.

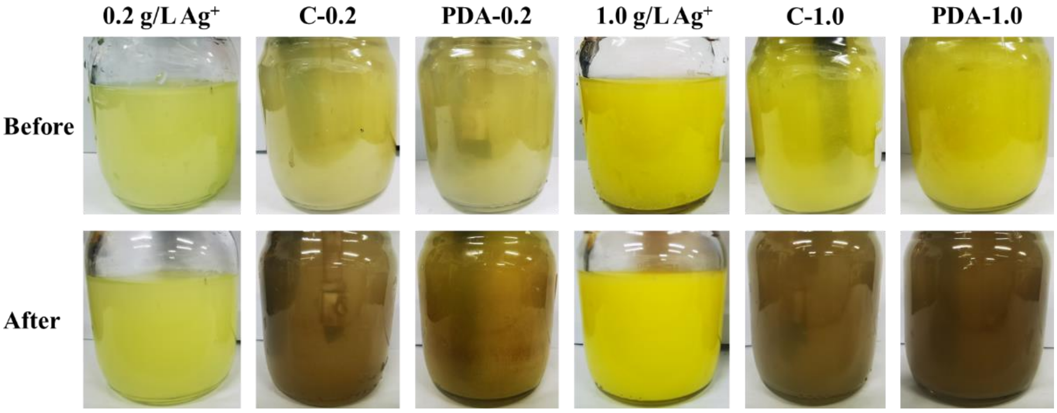

Figure S3. The digital photo of color change during the bioreduction of Ag^+. Related to Figure 2.

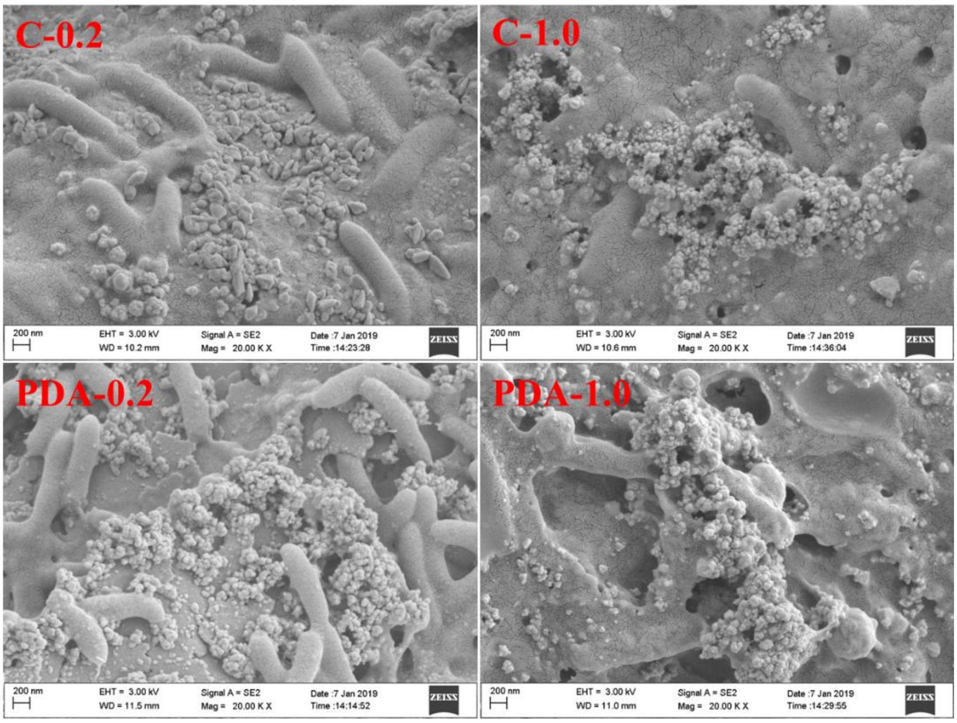

Figure S4. Scanning electron microscope (SEM) images of biofilm after bioreduction of Ag^+. Related to Figure 3.

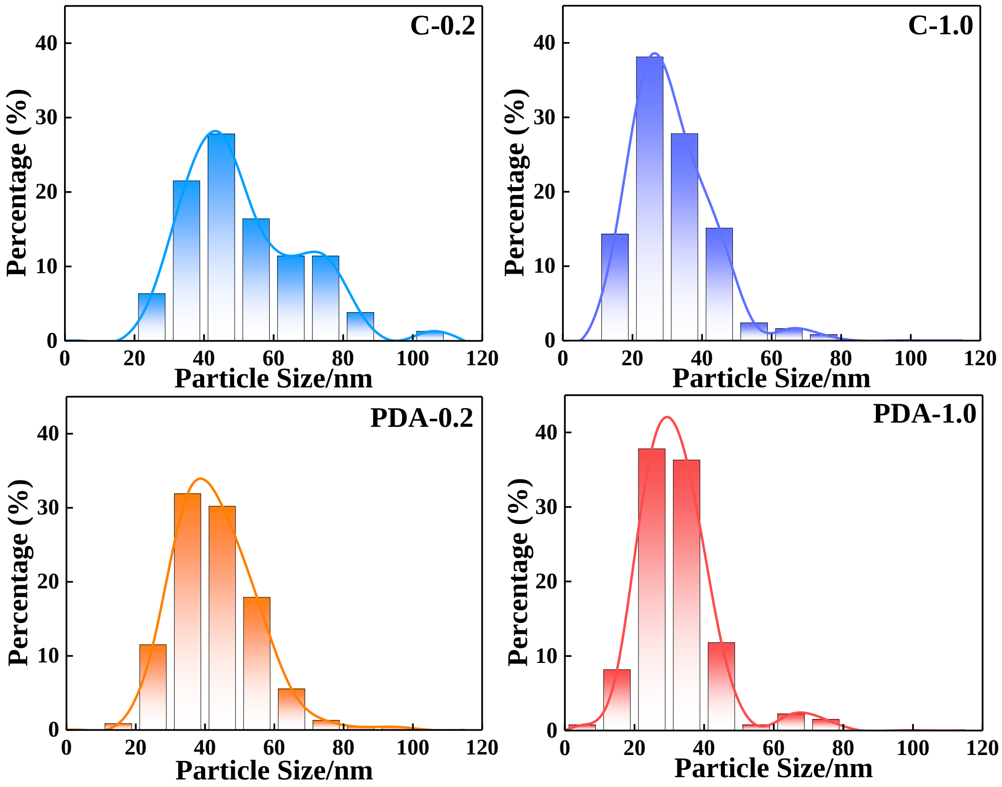

Figure S5. Size distribution of AgNPs formed by the bioreduction of Ag^+. Related to Figure 3.

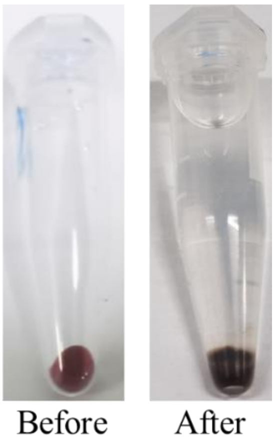

Figure S6. Photos of biofilms scraped from the graphite rod electrodes before and after Ag^{+} bioreduction. Related to Figure 4.

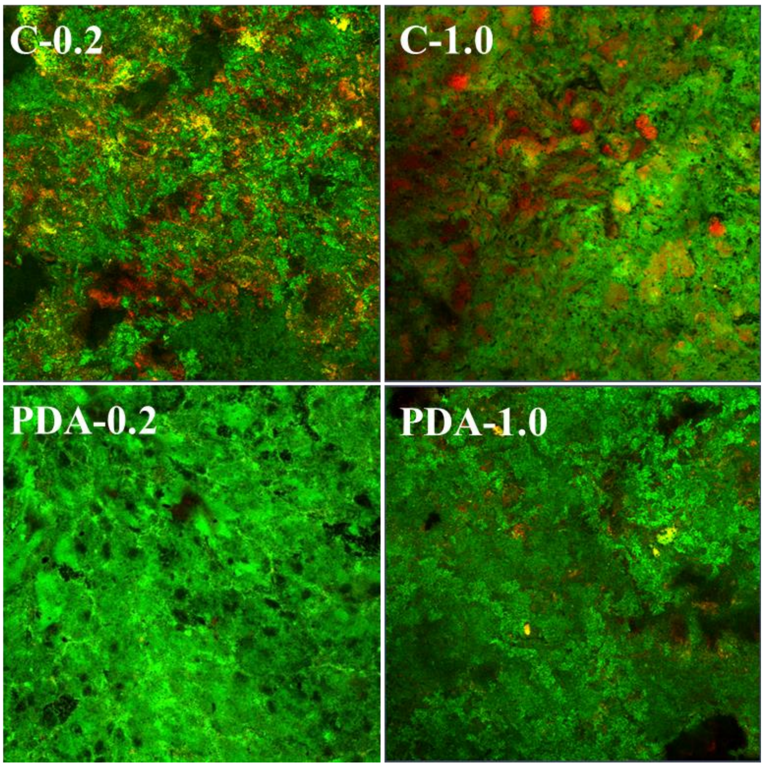

Figure S7. The 2D plan CLSM images of biofilm after Ag^{+} bioreduction. Related to Figure 4.

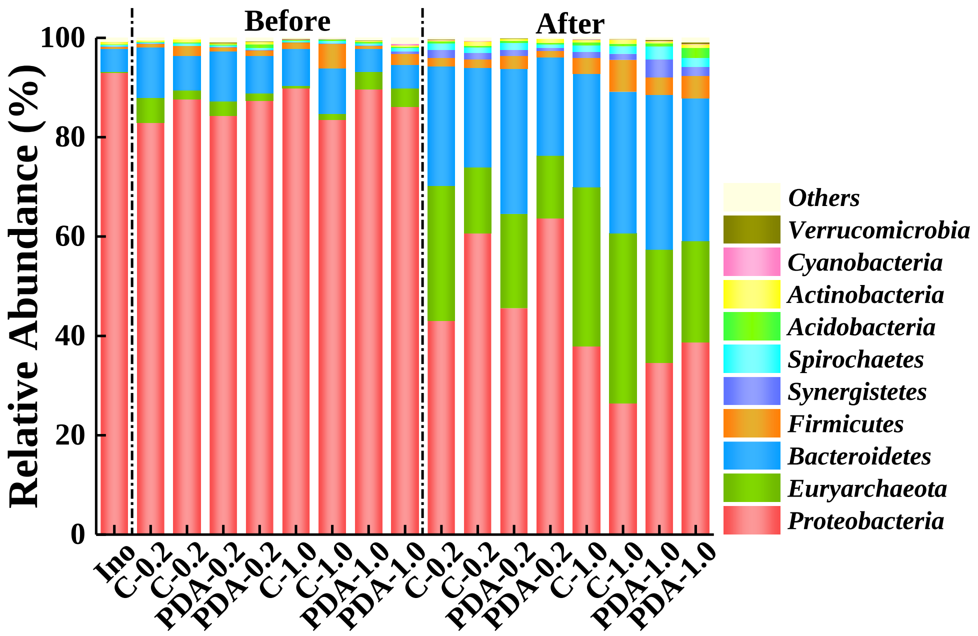

Figure S8. Relative abundance of bacterial communities at phylum level, two parallel samples were set for each group. Ino denotes inoculum and two identical pronouns denote samples removed from the parallel reactor. Related to Figure 5.

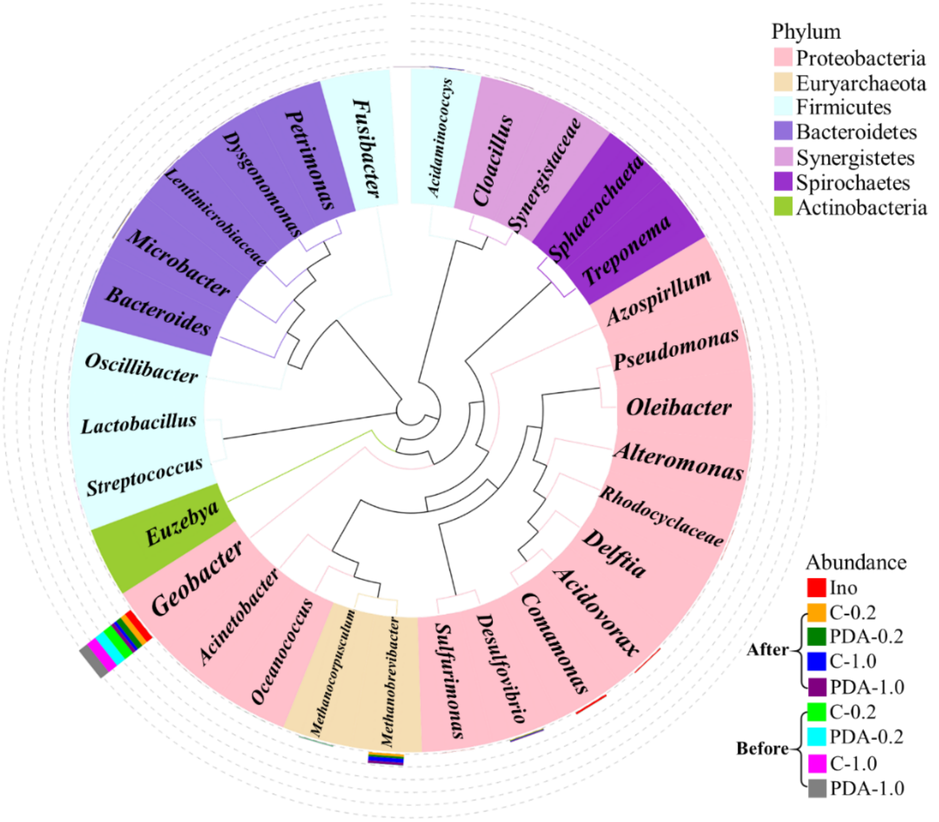

Figure S9. Phylogenetic relationships of species at genus level. Related to Figure 5.

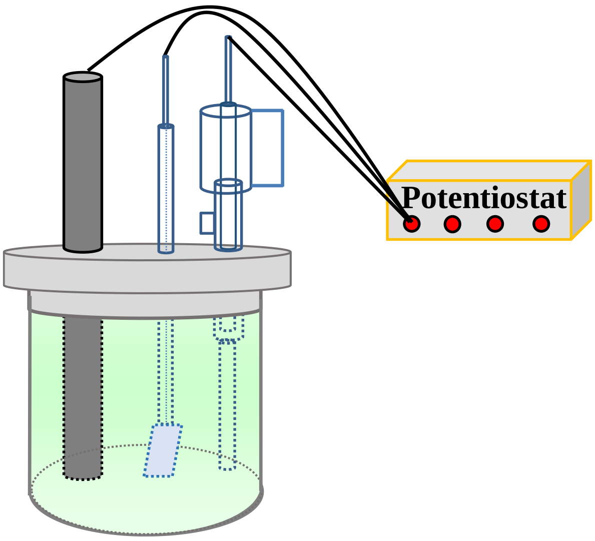

Figure S10. Schematic of three-electrode single chamber BESs bioreact. Related to STAR Methodes.
